# Magnetic nanoparticle-based method for microorganism concentration in sterile body fluids: Validation and clinical applications

**DOI:** 10.1007/s11274-025-04463-y

**Published:** 2025-07-01

**Authors:** Bilsen Tural, Erdal Ertaş, Nurullah Uzuner, Buşra Bektaş, Emre Tural, Mehmet Çavdar, Hakan Temiz, Erdal Özbek, Servet Tural

**Affiliations:** 1 Department of Nanotechnology, Institute of Science, Dicle University, 21280 Diyarbakir, Turkey; 2 Department of Chemistry, Institute of Science, Dicle University, 21280 Diyarbakir, Turkey; 3 Department of Food Processing, Technical Sciences Vocational School, Batman University, Batman, Turkey; 4 25 Aralık State Hospital Şehitkamil, Gaziantep, Turkey; 5 Faculty of Medicine, Department of Child Health and Diseases, Istanbul University-Cerrahpasa, Istanbul, Turkey; 6 Department of Medical Microbiology, Faculty of Medicine, Dicle University, Diyarbakir, Turkey

**Keywords:** Cerebrospinal fluid diagnostics, Clinical microbiology, Detection sensitivity, Low-density microorganisms, Magnetic nanoparticles, Microbial detection

## Abstract

Rapid and accurate detection of microorganisms in sterile body fluids, particularly cerebrospinal fluid (CSF), is crucial for effective diagnosis and treatment. Conventional methods, such as centrifugation, may result in low microbial recovery and false negatives, limiting diagnostic accuracy. An alternative, efficient, and accessible microbial concentration method is needed. This study evaluates a nanoparticle-based microbial concentration method to enhance pathogen recovery from CSF. The method was optimized for interaction time (1 min) and nanoparticle dosage (0.01 g/mL) using standard microbial strains, including *Staphylococcus aureus*, *Enterococcus faecalis*, *Escherichia coli*, *Pseudomonas aeruginosa*, and *Candida albicans*. Its clinical performance was assessed using 800 CSF samples, and microbial identification was confirmed via MALDI-TOF MS. The method significantly improved microbial recovery across 10^-2^–10^-9^ CFU/mL concentrations, achieving a detection limit as low as 2 CFU/mL. Clinical validation demonstrated 100% sensitivity and specificity, detecting 15 additional true-positive cases missed by centrifugation. While centrifugation fails to detect bacteria below 10^-7^ CFU/mL, our method reliably detects even at 10^-9^ CFU/mL, demonstrating superior microbial enrichment, especially in low-biomass samples. This method enhances diagnostic accuracy by reducing false negatives and expediting pathogen detection. Its resource-conscious, low-cost and equipment-free nature makes it particularly beneficial for resource-limited laboratories, offering a scalable alternative for microbial concentration in CSF diagnostics.

**Graphical Abstract:**

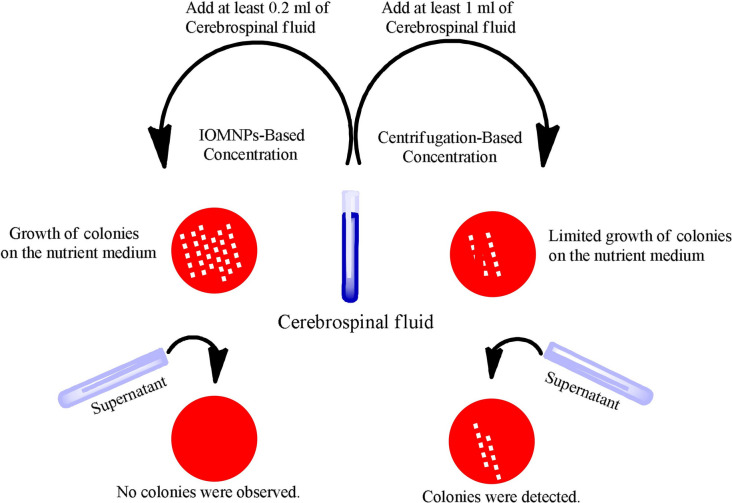

**Supplementary Information:**

The online version contains supplementary material available at 10.1007/s11274-025-04463-y.

## Introductıon

Cerebrospinal fluid (CSF) analysis is essential for diagnosing central nervous system (CNS) infections such as bacterial meningitis and encephalitis (Brouwer et al. 2010; Posnakoglou et al. 2020). Accurate and timely detection of microorganisms in CSF is critical, yet the inherently low microbial density in these samples often leads to false negatives using conventional methods (Deisenhammer et al. 2006; Heckenberg et al. 2014).

Centrifugation remains the standard technique for concentrating microorganisms in clinical microbiology laboratories. However, it is time-consuming, labor-intensive, and inconsistent, potentially failing to recover low-density pathogens (Alam et al. 2014; Enany et al. 2014). Molecular diagnostic methods, such as nucleic acid amplification tests, offer improved specificity but require advanced infrastructure and are costly, limiting their accessibility in resource-limited settings (Parkhe and Tiwari 2024). Therefore, a simple, low-cost, and equipment-independent method is necessary to enhance microbial detection in CSF samples.

Magnetic nanoparticles (MNPs) have been extensively explored in biomedical applications due to their high surface area, magnetic properties, and biocompatibility (Tural et al. 2024; Gupta and Gupta 2005). In microbial detection, functionalized MNPs selectively capture target pathogens, improving diagnostic sensitivity (Ai et al. 2024). However, such approaches are often limited to specific microorganisms, reducing their clinical applicability (Refai 2003). This study proposes an unmodified iron oxide magnetic nanoparticle (IOMNP)-based concentration method, allowing for cumulative microbial enrichment without requiring pathogen-specific modifications.

The primary objective of this study is to develop, optimize, and validate an IOMNP-based method for microbial concentration in CSF samples. This technique is compared to conventional centrifugation to assess its diagnostic performance, including sensitivity, specificity, and reproducibility. Additionally, the method’s applicability in resource-limited laboratories is evaluated, emphasizing its potential to replace existing labor-intensive techniques.

## Materıal and methods

### Chemicals and materials

All reagents used were of analytical grade. Iron oxide magnetic nanoparticles (IOMNPs) were synthesized using ferric chloride hexahydrate (FeCl_3_·6H_2_O) and ferrous chloride tetrahydrate (FeCl_2_·4H_2_O), both from Sigma-Aldrich. Ammonium hydroxide (NH_4_OH, 28–30%, Merck) was used as a precipitating agent in the co-precipitation method. Milli-Q ultrapure water was used for all synthesis and washing steps to ensure consistency. Standard microbial strains, including *Escherichia coli* (E. coli) (ATCC 25992), *Staphylococcus aureus* (S. aureus) (ATCC 13552), *Enterococcus faecalis* (E. faecalis) (ATCC 29212), *Pseudomonas aeruginosa* (P. aeruginosa) (ATCC 25853), and *Candida albicans* (C. albicans) (ATCC 10231), were obtained from a certified repository. Bacterial cultures were prepared in tryptic soy broth (TSB), nutrient broth (NB), and plated on nutrient agar (Oxoid) for enumeration.

### Synthesis and characterization of IOMNPs

IOMNPs were synthesized using the co-precipitation method, following established protocols (Laurent et al. 2010; Sun et al. 2008; Lu et al. 2007). Ferric and ferrous chloride salts (2:1 molar ratio) were dissolved in deoxygenated deionized water under an argon atmosphere to prevent oxidation. NH_4_OH (28%) was added dropwise under continuous stirring, leading to IOMNP precipitation. The reaction was refluxed at 80 °C for 30 min, and the nanoparticles were separated using a permanent magnet, washed with bidistilled water, and freeze-dried for stability. IOMNPs were characterized using transmission electron microscopy (TEM), scanning electron microscopy (SEM), dynamic light scattering (DLS), vibrating sample magnetometry (VSM), and Fourier-transform infrared spectroscopy (FT-IR) to evaluate their size, morphology, magnetic properties, and long-term stability over 18 months (Xu et al. 2019; Elahi and Rizwan 2021; Berry and Curtis 2003). IOMNPs were suspended in deionized water (50 mL total volume), homogenized using an ultrasonic processor, and sterilized at 120 °C for 15 min in an autoclave before each experiment. Stability assessments were conducted at three-month intervals for 18 months, measuring DLS, zeta potential, and VSM. FT-IR analysis confirmed structural integrity.

### Microbial strains and sample preparation

Bacterial suspensions were prepared at 0.10–0.12 McFarland units in ID broth and serially diluted to 10^−2^–10^−9^ CFU/mL (Alam et al. 2014; Prabhurajeshwar and Chandrakanth 2019; Schön et al. 2020). A 0.01 mL aliquot of each dilution was plated on 5% sheep blood agar (SBA) for bacterial strains or Sabouraud Dextrose Agar (SDA) for *C. albicans*. For comparative analysis, samples were processed using two different concentration methods. In the centrifugation method, samples were spun at 2000 rpm for 2 min (Tunkel et al. 2004). In the IOMNP-based method, 1 mL of the sample was mixed with 100 µL of IOMNP suspension (1 mg/mL) and incubated for 1 min at room temperature with gentle agitation. The IOMNP-microorganism complexes were then separated using a neodymium magnet, and the resulting pellet was resuspended in 100 µL of sterile PBS before plating. Negative controls, consisting of sterile IOMNP suspensions without bacterial inoculation, were included to confirm method sterility.

### Clinical CSF samples

A total of 800 cerebrospinal fluid (CSF) samples were obtained from surplus clinical specimens processed in the microbiology laboratory at Dicle University Medical Faculty Hospital. These samples were derived from routine diagnostic procedures performed on patients from various departments, including neurology, pediatric emergency, intensive care, and neurosurgery. After diagnostic analyses, the remaining CSF samples, with a minimum volume of 2 mL, were anonymized and used for this study. Each sample was divided equally: 1 mL was processed using the conventional centrifugation method, and 1 mL was processed using an iron oxide magnetic nanoparticle (IOMNPs)-based concentration method. The IOMNPs-based method involved mixing 1 mL of CSF with 100 µL of IOMNP suspension (1 mg/mL) and incubating at room temperature for 10 min with gentle agitation. The mixture was magnetically separated, and the concentrated microbial pellet was resuspended in 100 µL of sterile phosphate-buffered saline (PBS). The diagnostic performance of both methods was evaluated and compared based on culture results. Pathogens isolated from CSF samples were identified using a MALDI-TOF MS system (Bruker Microflex LT, Bruker Daltonics, Germany) operated in linear positive mode with a laser frequency of 60 Hz. Single colonies were picked from culture plates, prepared via the ethanol/formic acid extraction method (10 µL of ethanol/formic acid solution per colony), and analyzed with MALDI-TOF MS. Mass spectra were matched against the Bruker Biotyper database, and species-level identification was confirmed for scores of ≥ 2.0 (Tunkel et al. 2004; Wattal et al. 2017). This method enabled the rapid and accurate identification of a broad range of clinically relevant pathogens. To ensure sterility, blank plating was performed using IOMNPs without clinical CSF samples. No microbial growth was observed, verifying the sterility of the reagents and the accuracy of the methods. All procedures were conducted in a Class 2 biosafety cabinet to minimize contamination risks. The study complied fully with ethical standards. No specific patient samples were collected for this research; instead, surplus cerebrospinal fluid samples from routine diagnostic procedures in the microbiology laboratory were anonymized and used. Furthermore, no patient-identifiable information was collected or utilized at any stage of the study. The study complied fully with ethical standards.

### Statistical analysis

All experiments were conducted in triplicate, and data are presented as mean ± standard deviation (SD). Normality was assessed using the Shapiro–Wilk test, while differences among groups were evaluated using the Kruskal–Wallis test. For pairwise comparisons, the Wilcoxon signed-rank test was applied for non-parametric data, whereas independent t-tests or ANOVA were used for normally distributed datasets. Levene’s test was conducted to assess variance homogeneity, followed by either Tukey’s HSD or Games-Howell post hoc tests, depending on variance assumptions. Statistical significance was set at *p* < 0.05, and all analyses were performed using IBM SPSS Statistics 24.0. Figures were generated using GraphPad Prism 9.0 (Salmeron et al. 2022).

## Results

### Characterization of IOMNPs

The morphological and structural properties of IOMNPs were characterized using TEM, SEM, and FTIR analyses, as shown in Figure S1. These analyses confirmed the uniform size distribution, crystalline structure, and functional groups of IOMNPs, supporting their suitability for microbial concentration.

To evaluate microbial attachment, FT-IR spectra of IOMNPs after interaction with *E. coli, S. aureus, P. aeruginosa, C. albicans,* and *E. faecalis* were obtained. The spectra of bare IOMNPs and microorganism-attached IOMNPs were compared (Figure S2). Distinct spectral shifts and additional absorption bands indicated successful microbial adhesion to IOMNP surfaces, highlighting the physicochemical interactions responsible for microbial capture. These observations are consistent with previous studies demonstrating the role of nanoparticle surface properties and biomolecular interactions in microbial binding (Velmathi et al. 2024; Abarca-Cabrera et al. 2021).

### Stability analysis of IOMNPs

**Fig. 1:**
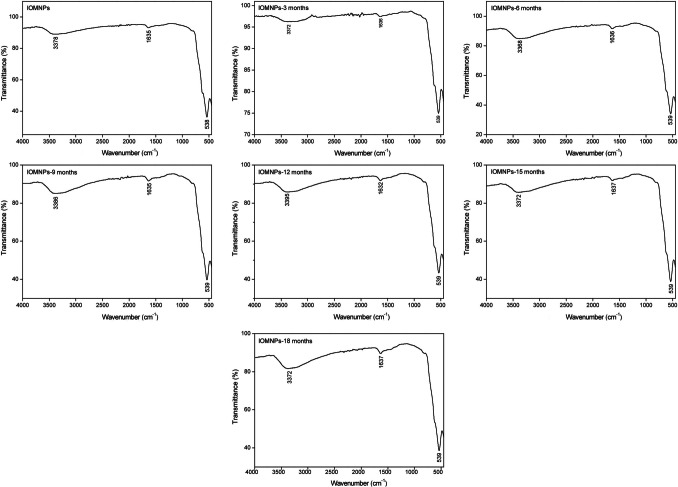
FT-IR spectra of IOMNPs obtained at 3-month intervals over an 18-month period

FT-IR spectral analysis (Fig. 1) confirmed the structural stability of IOMNPs over an 18-month storage period. The Fe–O stretching vibration bands at 500–600 cm^−1^, indicative of the spinel structure of magnetite, remained unchanged, demonstrating preservation of the Fe_3_O_4_ lattice (Cornell and Schwertmann 2003; Goya et al. 2003). Additionally, the broad absorption bands at 3300–3700 cm^−1^, corresponding to surface hydroxyl groups and adsorbed water molecules, exhibited no significant changes, confirming stable surface hydration and resistance to structural degradation (Sacko et al. 2024).

Magnetic stability, assessed using VSM (Figure S3, Table 1), showed a slight decrease in saturation magnetization (Ms) from 68.42 emu/g to 63.92 emu/g after 18 months, attributed to partial oxidation or agglomeration (Gupta and Gupta 2005; He et al. 2014). Despite this reduction, Ms values remained above 50 emu/g, sufficient for effective magnetic separation (Laurent et al. 2008). Both coercivity (Hc) and remanent magnetization (Mr) remained stable throughout the study, with Hc approximately 50 Oe, indicative of consistent superparamagnetic behavior (Iida et al. 2007).

**Table 1:** Changes in zeta potential, particle size, and magnetic stability (saturation magnetization) of IOMNPs over an 18-month period

Time Point	Z-Average (nm)	Zeta Potential (mV)	Saturation Magnetization (emu/g)
Fresh	50.79	−31.85	68.42
3 months	68.69	−23.10	68.05
6 months	79.88	−21.97	66.42
9 months	92.89	−19.79	65.70
12 months	108.00	−16.44	64.65
15 months	125.60	−15.48	64.41
18 months	203.20	−13.88	63.92

DLS analysis (Figure S4, Table 1) revealed an initial narrow size distribution of 50–100 nm, which increased to 200–400 nm after 18 months, likely due to particle agglomeration. This increase corresponds with a reduction in electrostatic repulsion as surface charge decreased over time (Laurent et al. 2008). Despite this, the functional performance of IOMNPs in magnetic separation remained unaffected.

Zeta potential measurements (Figure S5, Table 1) initially showed a value of −31.85 mV, indicating strong electrostatic repulsion and colloidal stability. Over 18 months, the zeta potential gradually decreased to −13.88 mV, reflecting surface charge neutralization and particle agglomeration. However, values remained above the critical stability threshold (± 10–15 mV), ensuring sufficient dispersibility for practical applications (Vohl et al. 2024).

### Studies with standard microbial strains

#### Optimization of ıncubation time

The impact of incubation time on microbial concentration efficiency using the IOMNP-based method was evaluated. Raw colony count data (Table S1) were analyzed statistically, with summarized results presented in Table 2. Bacterial suspensions of *E. coli*, *S. aureus*, *P. aeruginosa*, *C. albicans*, and *E. faecalis* at 10^−7^ CFU/mL were incubated for 1, 15, 30, 60, and 180 min. The Kruskal–Wallis test indicated no statistically significant differences in colony counts across time intervals (*p* > 0.05 for all strains). Despite minor variations, 1-min incubation consistently yielded rapid and reliable microbial recovery across all tested species, supporting its use as the optimal incubation duration (Kruskal and Wallis 1952).

**Table 2:** Kruskal–Wallis Test results for colony counts based on time for different microorganisms

Bacterial Strain	H-Statistic	*p*-Value	Result	Decision
*S. aureus*	6.511432	0.164071	No Significant Difference	1 min is suitable
*E. coli*	5.555145	0.234925	No Significant Difference	1 min is suitable
*E.faecalis*	7.292229	0.121228	No Significant Difference	1 min is suitable
*P.aeruginosa*	0.524155	0.971107	No Significant Difference	1 min is suitable
*C.albicans*	6.898917	0.141327	No Significant Difference	1 min is suitable
Overall	1.574567	0.813356	No Significant Difference	1 min is suitable

#### Optimization of IOMNPs dosage

The optimal IOMNP dosage for microbial concentration was evaluated by testing four concentrations (0.007 g/mL, 0.01 g/mL, 0.015 g/mL, and 0.02 g/mL) on standard bacterial strains, including *S. aureus*, *E. coli*, *P. aeruginosa*, *C. albicans*, and *E. faecalis*. The results (Table S2, Table 3) indicated that 0.01 g/mL IOMNPs consistently produced the highest colony counts with minimal standard deviation, demonstrating both efficiency and reproducibility. Lower concentrations, such as 0.007 g/mL, exhibited slightly variable results, likely due to insufficient nanoparticle availability for consistent bacterial capture. In contrast, higher concentrations (0.015 g/mL and 0.02 g/mL) resulted in reduced colony counts and greater variability, attributed to nanoparticle agglomeration, which decreases the effective surface area for microbial binding. Statistical analysis using the Kruskal–Wallis test did not reveal significant differences between doses (*p* = 0.098); however, Cliff’s Delta analysis indicated a moderate effect size (Δ = 0.462) favoring 0.01 g/mL IOMNPs as the optimal concentration.

**Table 3:** Statistical analysis results for dose study across all bacteria

Test Type	Data Used	Statistical Value	*p*-Value	Comment
Shapiro–Wilk	All bacteria, all doses	W = 0.692	*p* < 0.05	Data are not normally distributed
Kruskal–Wallis	All bacteria, all doses	H = 6.30	*p* = 0.098	No significant differences among doses
Cliff's Delta	7 mg vs 10 mg	Δ = 0.462	—	Moderate effect size, 10 mg performed better

### Studies with standard microbial strains

#### The effect of microorganism concentration on the efficiency of enrichment methods

The efficiency of three microbial recovery methods (Standard Plating, Centrifugation-Based Concentration, and IOMNPs-Based Concentration) was evaluated for *S. aureus*, *E. coli*, *E. faecalis*, *P. aeruginosa*, and *C. albicans* across bacterial concentrations ranging from 10^−2^ to 10^−9^ CFU/mL. The IOMNPs-based method demonstrated superior performance compared to both standard plating and centrifugation, particularly at low bacterial densities (Table S3).

Standard Plating method failed to detect bacteria below 10^−5^ CFU/mL for most strains. *S. aureus* and *P. aeruginosa* were undetectable at 10^−7^ and 10^−8^ CFU/mL, respectively, underscoring its limited sensitivity in detecting sparse bacterial populations (Table S3) (Kulkarni et al. 2023).

While centrifugation-based concentration improved bacterial recovery compared to standard plating, particularly at intermediate levels (e.g., 10^−6^ CFU/mL), it exhibited high variability and reduced efficiency at concentrations below 10^−8^ CFU/mL, particularly for *E. coli* (Table S3).

The IOMNPs-based method demonstrated exceptional sensitivity and reproducibility, successfully recovering bacterial loads as low as 10^−9^ CFU/mL for *E. faecalis* and *P. aeruginosa*. These findings align with prior studies demonstrating the high capture efficiency of magnetic nanoparticles (Table S3) (Li et al. 2019).

##### Statistical validation

The Kruskal–Wallis test revealed significant differences among the three microbial recovery methods (*p* < 0.05). Post-hoc comparisons confirmed that the IOMNPs-based method was significantly superior to both standard plating and centrifugation-based concentration. Effect size analysis using Cohen’s d indicated moderate superiority of IOMNPs-based concentration over standard plating (d = 0.56) and small superiority over centrifugation-based concentration (d = 0.27) (Table 4) (Takallu et al. 2024). In addition to sensitivity and specificity, we also calculated Cohen’s Kappa coefficients to evaluate the agreement between each enrichment method and standard culture results. The IOMNP-based method demonstrated a Kappa value of 1.000, indicating perfect agreement. In contrast, the centrifugation method yielded a Kappa value of 0.874, corresponding to almost perfect agreement. These results support the superior consistency of the IOMNP-based approach in detecting viable microorganisms.

**Table 4:** Comparison of the effectiveness of standard plating, centrifugation-based concentration, and IOMNPs-based concentration using Cohen’s d effect size

Comparison of Methods	Mann–Whitney U Test Statistic	*p*-Value	Cohen’s d Value	Effect Size Level
IOMNPs-based vs Standard Plating	1848	0.00038	0.56	Medium Effect
IOMNPs-based vs Centrifugation-based	1690.5	0.00623	0.27	Small Effect

##### Visual representation

**Fig. 2:**
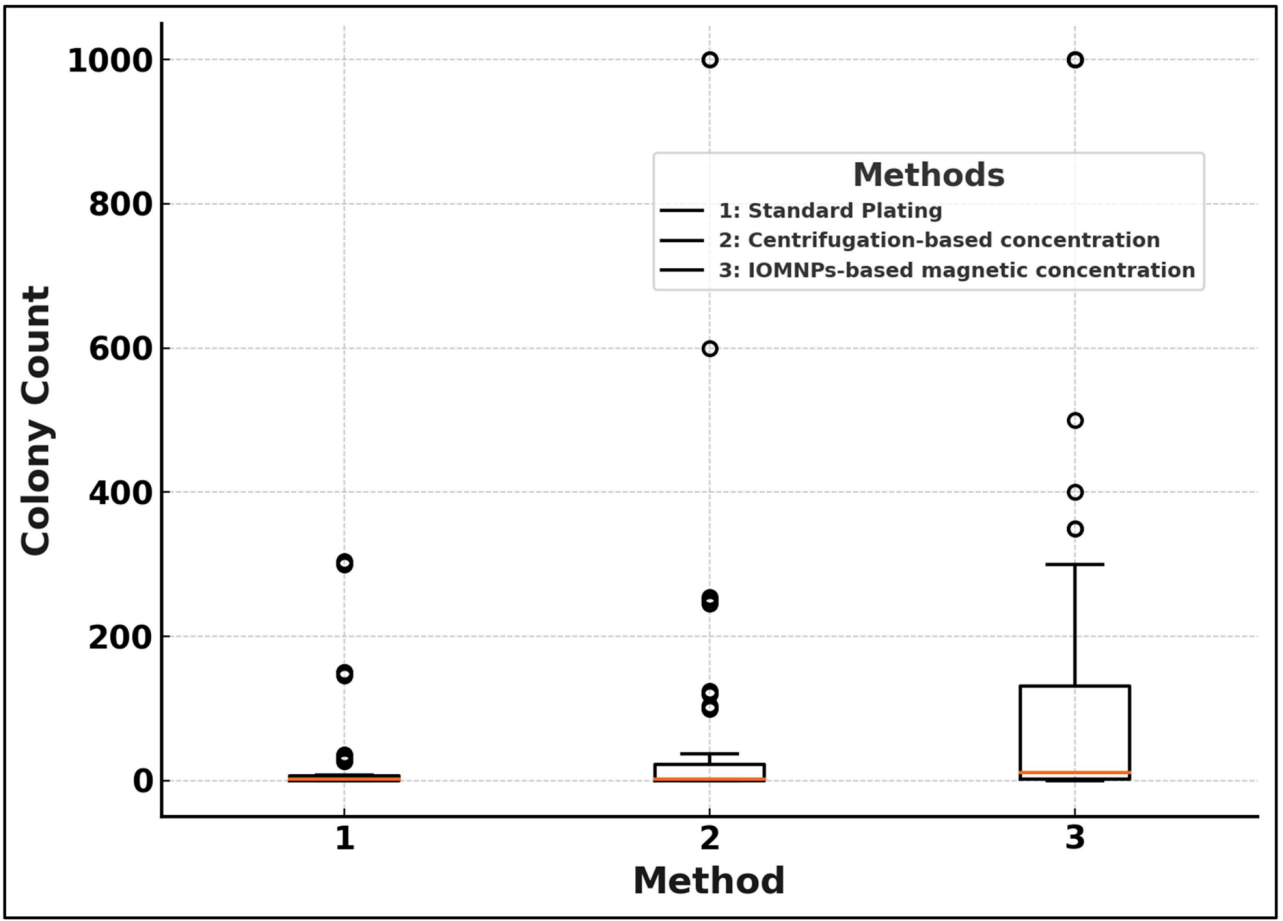
Boxplot for General Bacteria: Overall comparison of the effectiveness of methods for all microorganisms. (1: Standard Plating Method, 2: Centrifugation-Based Concentration and 3: IOMNPs-Based Concentration)

Boxplots (Figure S6–S10) illustrated the variability and median values for each method. The IOMNPs-based method exhibited the highest median colony counts and the narrowest interquartile ranges, indicating high sensitivity and consistency. Figure 2 summarizes the collective performance of all three methods across the tested microorganisms, emphasizing the superiority of IOMNPs-based concentration in recovering low-density bacteria from CSF samples.

##### Limit of detection (LOD) for IOMNPs-based concentration method

The IOMNPs-based concentration method was evaluated for its limit of detection (LOD) across various bacterial concentrations, as summarized in Table S4. The LOD values (Table 5) highlight the method’s high sensitivity. For *P. aeruginosa* and *E. faecalis*, the LOD reached 10^−9^ CFU/mL, demonstrating exceptional detection capability. In contrast, *C. albicans* exhibited a higher LOD of 10^−7^ CFU/mL, likely due to its larger cell size and lower magnetic affinity. These findings emphasize the ability of the IOMNPs-based method to detect microorganisms at extremely low concentrations, underscoring its suitability for sterile body fluids, such as CSF.

**Table 5:** Limit of detection (LOD) values and average colony counts using IOMNPs-based concentration method

Bacterial Strain	Concentration (CFU/mL)	Average Colony Count	Standard Deviation	Minimum Colony Count	Maximum Colony Count	LOD (≥ 2 Colonies)
*S. aureus*	10^−7^	67.7	2.5	65	70	TRUE
*S. aureus*	10^−8^	2.3	0.6	2	3	TRUE
*E. coli*	10^−7^	48.7	22.9	33	75	TRUE
*E. coli*	10^−8^	5.0	3.6	2	9	TRUE
*E. faecalis*	10^−7^	145.0	18.0	125	160	TRUE
*E. faecalis*	10^−8^	13.3	4.2	10	18	TRUE
*E. faecalis*	10^−9^	2.3	0.6	2	3	TRUE
*P. aeruginosa*	10^−7^	161.7	37.5	125	200	TRUE
*P. aeruginosa*	10^−8^	10.3	2.5	8	13	TRUE
*P. aeruginosa*	10^−9^	2.3	1.52	1	4	TRUE
*C. albicans*	10^−7^	2.0	1.0	1	3	TRUE

### Iomnps-based concentration of microorganisms in clinical CSF samples

The performance of IOMNPs-based and centrifugation-based methods for microorganism enrichment in clinical CSF samples was compared. A total of 800 CSF samples were processed using both methods. The standard plating method was excluded due to its previously demonstrated low diagnostic sensitivity with five standard microbial strains.

**Fig. 3:**
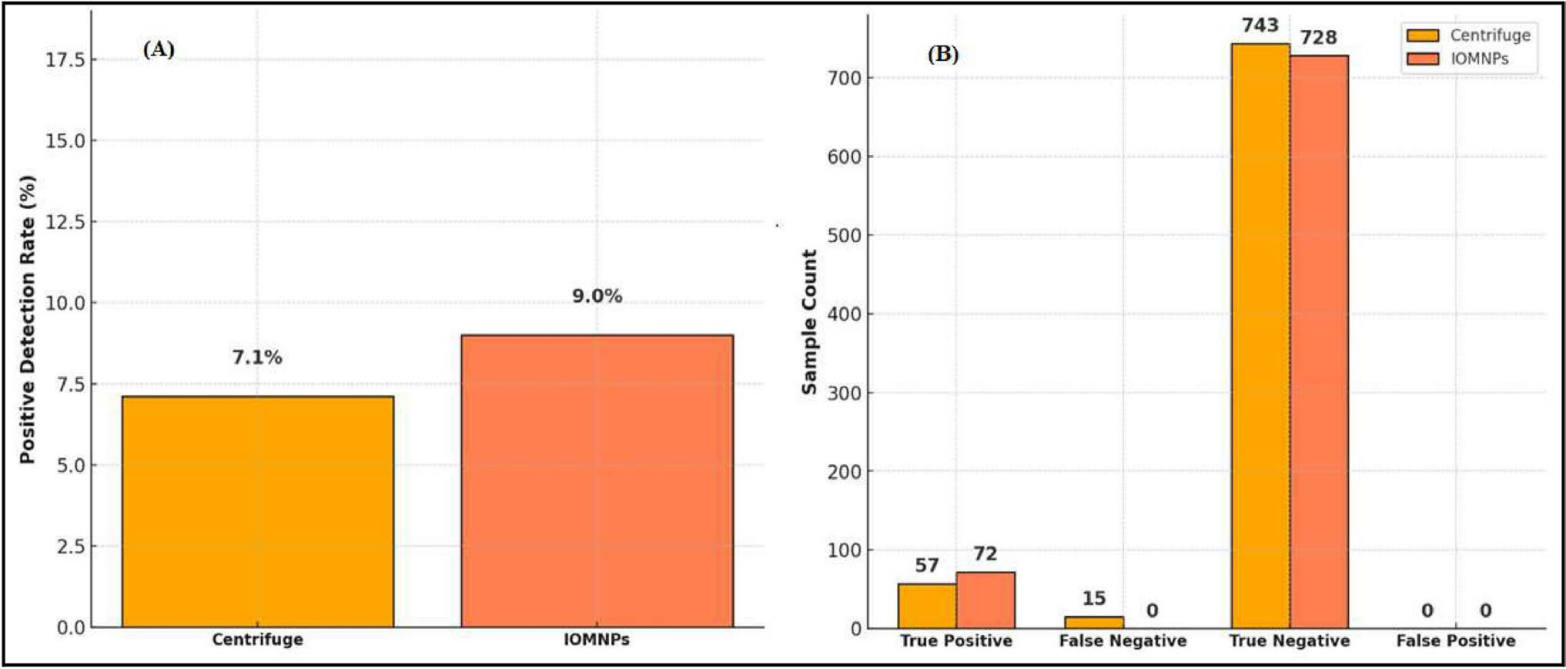
**A** Positive detection rates for centrifugation and IOMNPs methods, **B** Diagnostic outcomes comparison: True/False Positives and Negatives

The IOMNPs-based method exhibited significant advantages over centrifugation, achieving a higher detection rate of 9.0% compared to 7.1% for centrifugation (Table 6, Fig. 3A). The IOMNPs-based method detected 72 true positives (TP) without any false negatives (FN = 0), whereas centrifugation identified 57 true positives but missed 15 cases (FN = 15). Both methods showed high true-negative rates, with 743 for centrifugation and 728 for IOMNPs, and no false positives (FP = 0) were observed (Fig. 3B, Table 6).

**Table 6:** Comparison of sensitivity, specificity, and accuracy for centrifugation and IOMNP-based methods

Method	True Positive (TP)	False Negative (FN)	True Negative (TN)	False Positive (FP)	Sensitivity (%)	Specificity (%)	Accuracy (%)
Centrifugation	57	15	728	0	79.2	100	98.1
IOMNPs	72	0	728	0	100	100	100

Sensitivity analysis showed that the IOMNPs-based method achieved a sensitivity of 100%, significantly outperforming centrifugation (79.2%). Specificity was equally high for both methods (100%). The IOMNPs-based method also exhibited perfect accuracy (100%), surpassing centrifugation (98.1%). Negative control tests confirmed the efficiency of the IOMNPs-based process, as no bacterial growth was detected in the supernatants.

#### Pathogen ıdentification

**Fig. 4:**
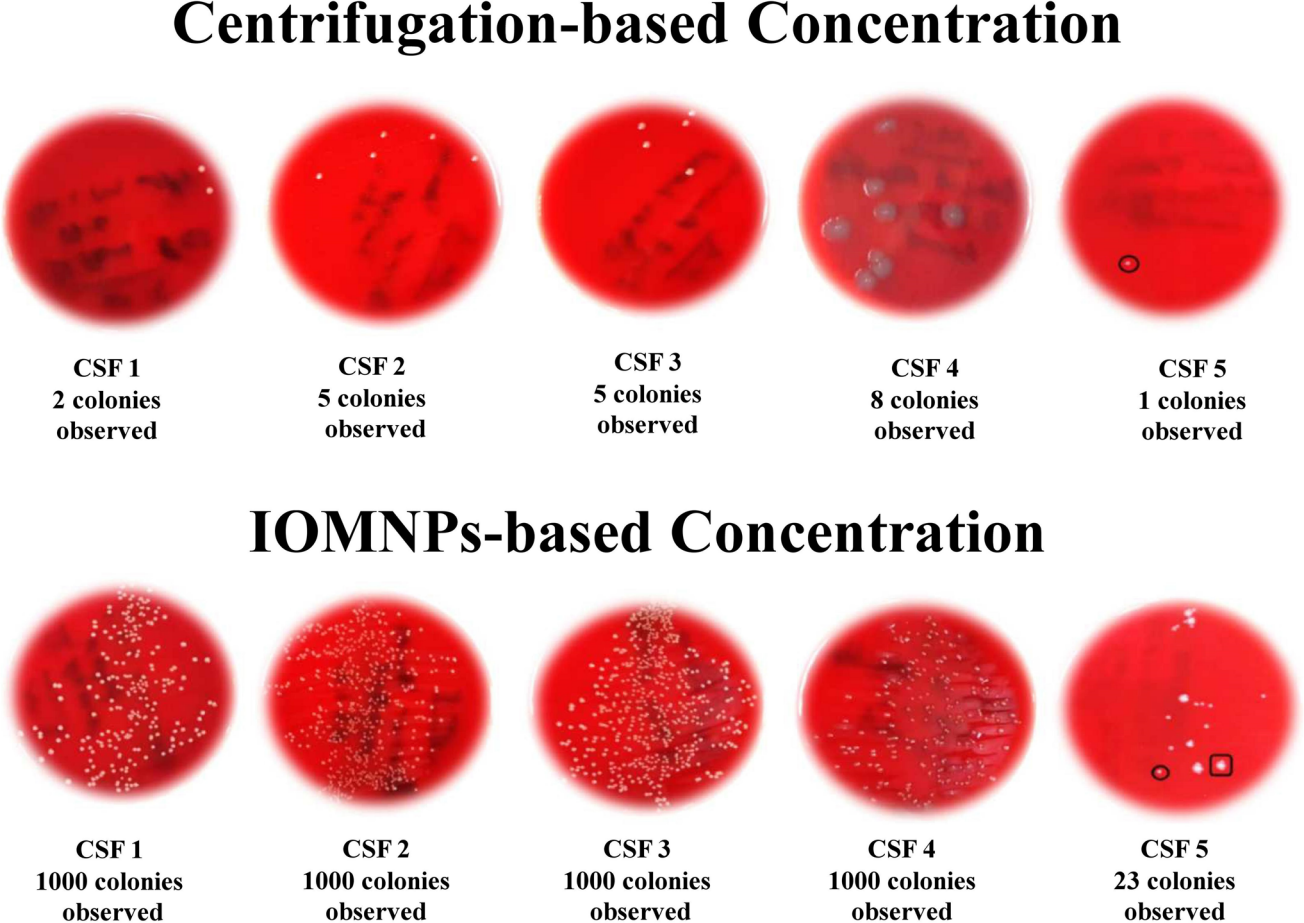
Plate images and colony counts of five clinical CSF samples processed using centrifugation-based and IOMNPs-based concentration methods

The findings in Fig. 4 and Figure S11 highlight the superior performance of the IOMNPs-based concentration method over centrifugation in processing CSF samples. In five CSF samples where both methods detected microorganisms, the IOMNPs-based method consistently yielded significantly higher colony counts, reaching up to 1000 CFU/mL, compared to the centrifugation method’s range of 2–8 CFU/mL. Additionally, no microbial growth was detected in the supernatants of the IOMNPs-based method, confirming its efficiency in fully capturing microorganisms. In contrast, centrifugation exhibited residual microbial growth in the supernatants, underscoring its limitations.

MALDI-TOF MS analysis of the captured pathogens identified a broad spectrum of clinically relevant species frequently associated with CSF infections, including *Serratia marcescens* (2.2), *Acinetobacter baumannii* (2.3), *Klebsiella pneumoniae* (2.1), *Streptococcus pyogenes* (2.4), *Streptococcus pneumoniae* (2.3), *Haemophilus influenzae* (2.1), *Neisseria meningitidis* (2.5), *Pantoea agglomerans* (2.2), and the fungal pathogen *Cryptococcus neoformans* (2.0). MALDI-TOF MS scores (≥ 2.0) confirmed species-level identification for all isolates, further validating the diagnostic reliability of the IOMNPs-based method.

## Dıscussıon

This study introduces an innovative iron oxide magnetic nanoparticle (IOMNP)-based method for microbial concentration in CSF samples, demonstrating superior sensitivity compared to centrifugation, particularly for low-density microorganisms. However, the study has certain limitations that should be considered.

One limitation is the scope of microbial species evaluated. While a diverse set of bacteria and fungi were tested, additional validation with a broader spectrum of clinically relevant microorganisms would enhance the generalizability of the findings. Furthermore, certain pathogens, such as *Candida albicans*, exhibited higher limits of detection (LOD) due to larger cell size and lower magnetic affinity, suggesting a need for further optimization. Another limitation is that the study focused exclusively on CSF samples. Expanding the method’s application to other sterile body fluids, such as peritoneal or synovial fluids, could provide additional insights into its diagnostic potential (Cornell and Schwertmann 2003; Sacko et al. 2024; Laurent et al. 2008).

Despite these limitations, this study offers several key advantages over existing methods. Unlike previous approaches that rely on functionalized nanoparticles targeting specific pathogens, this method employs unmodified IOMNPs, allowing for cumulative microbial concentration across a broad range of species. This eliminates the need for surface modifications, reducing time and cost, while extending the method’s applicability in diverse clinical scenarios (Cornell and Schwertmann 2003; Sacko et al. 2024; Laurent et al. 2008). Recent studies on nanoparticle-based antimicrobial strategies have further emphasized the potential of such materials in biomedical applications, particularly in inhibiting microbial biofilm formation and improving pathogen recovery (Mustafa et al. 2021). Additionally, the method’s rapid processing time (1 min) and exceptional sensitivity (LOD as low as 10^−9^ CFU/mL for certain species) mark a substantial improvement over traditional techniques. Clinical validation demonstrated 100% sensitivity, specificity, and accuracy, addressing a critical gap in conventional diagnostic workflows, where low microbial density often results in false negatives (Sacko et al. 2024; Iida et al. 2007). These findings align with recent advances in biosensor-based rapid pathogen detection technologies, which emphasize the need for highly sensitive and rapid diagnostic platforms (Chen et al. 2018).

The high microbial capture efficiency of IOMNPs is attributed to multiple physicochemical and magnetic interactions. Van der Waals forces facilitate short-range physical attractions between nanoparticles and microbial surfaces, enabling efficient attachment despite electrostatic repulsion. Ionic screening in the solution further mitigates repulsive forces, particularly in high ionic strength media, enhancing nanoparticle-microbe interactions (Li et al. 2019; Cotin et al. 2018).

The magnetic properties of IOMNPs further enhance their capture efficiency. External magnetic field application brings nanoparticles and microorganisms into close proximity, improving attachment rates, a phenomenon consistently observed in studies on magnetic nanoparticles in biological matrices (Li et al. 2019; Kim et al. 2020). Additionally, hydroxyl groups on IOMNP surfaces facilitate hydrogen bonding with microbial cell wall components, stabilizing the nanoparticle-microbe complex. Spectral analysis further supports this hydrogen bonding mechanism, reinforcing the reproducibility of microbial capture (Iida et al. 2007; Boaretti et al. 2016). Another contributing factor is the nanoscale roughness of IOMNP surfaces, which enhances physical compatibility with microbial cell walls. Surface topography studies confirm that roughened surfaces provide additional attachment points, strengthening interactions (Li et al. 2019).

The long-term stability of the IOMNPs-based method over 18 months underscores its practical utility. Structural and magnetic integrity, including consistent FT-IR spectra and sufficient saturation magnetization values, ensured reliable performance over time (Li et al. 2019; Cotin et al. 2018; Kim et al. 2020). Although agglomeration and a reduction in zeta potential were observed, these changes did not compromise method effectiveness. Furthermore, while the study primarily focused on five microbial strains (*E. coli, S. aureus, E. faecalis, P. aeruginosa,* and *C. albicans*), MALDI-TOF MS analysis confirmed that the method efficiently concentrated other clinically relevant microorganisms, reinforcing its broad-spectrum diagnostic potential (Li et al. 2019; Cotin et al. 2018).

Compared to centrifugation, the IOMNPs-based method provided a clear diagnostic advantage, detecting 15 additional true-positive cases that were missed by the conventional approach. This ability to eliminate false negatives is particularly crucial for detecting central nervous system infections, where timely and accurate diagnosis is essential (Li et al. 2019; Helm et al. 1991). The statistical evaluation further supports the diagnostic consistency of the IOMNP-based method. While the centrifugation approach demonstrated almost perfect agreement with culture results (Cohen’s Kappa = 0.874), the IOMNP-based method showed perfect agreement (Cohen’s Kappa = 1.000). According to the Landis and Koch scale, values between 0.81 and 1.00 indicate almost perfect to perfect agreement (Landis and Koch 1977). These findings underscore the robustness of the IOMNP-based strategy, especially in detecting low-abundance microorganisms where minor enrichment losses may compromise diagnostic accuracy. This is particularly relevant for cases where conventional methods yield culture-negative results, underscoring the importance of alternative diagnostic markers in clinical microbiology (Boaretti et al. 2016).

In conclusion, while this study acknowledges certain limitations, the IOMNPs-based concentration method presents a transformative approach to microbial diagnostics. By combining broad-spectrum applicability, rapid processing, and superior sensitivity, this method offers a promising solution for detecting low-density microorganisms. Future research should focus on expanding its application to other biological fluids, optimizing its performance for larger or less magnetically responsive pathogens, and integrating it into automated diagnostic platforms to further enhance clinical utility (Li et al. 2019; Cotin et al. 2018; Kim et al. 2020).

## Conclusıon

This study optimized and evaluated the use of iron oxide magnetic nanoparticles (IOMNPs) for microbial concentration in clinical CSF samples. Compared to centrifugation, a conventional microbiology method, the IOMNPs-based concentration method demonstrated superior diagnostic performance, achieving 100% sensitivity and accuracy, while centrifugation achieved 79.2% sensitivity and 98.1% accuracy. The elimination of 15 false-negative results highlights the robustness of IOMNPs in detecting microorganisms, even at extremely low concentrations.

Long-term stability assessments conducted over 18 months at three-month intervals confirmed the structural and magnetic integrity of IOMNPs. Zeta potential measurements, DLS particle size distributions, and magnetic property analyses (VSM) revealed consistent performance without significant degradation, supporting the reliability and reproducibility of this method.

Under optimized conditions (1-min vortexing with a 0.01 g/mL IOMNPs concentration), the method achieved the highest recovery rates across five standard microbial strains (*E. coli, S. aureus, P. aeruginosa, E. faecalis,* and *C. albicans*). Detection limit experiments established thresholds as low as 2 CFU/mL, with *P. aeruginosa* and *E. faecalis*detected at 10^−9^ CFU/mL, underscoring the exceptional sensitivity of this method for low microbial loads.

Beyond its application to the tested bacterial strains, the IOMNPs-based method successfully captured additional clinically relevant pathogens, including *Neisseria meningitidis, Streptococcus pneumoniae,* and *Cryptococcus neoformans*. Integration with MALDI-TOF MS enhanced its versatility for multi-pathogen detection, making it a valuable tool for comprehensive diagnostics.

The proven stability, sensitivity, and adaptability of the IOMNPs-based method establish it as a robust alternative to traditional concentration techniques. Future research should explore its applicability to other biological fluids, expand its pathogen detection capabilities, and integrate it into routine diagnostic workflows, potentially improving clinical outcomes and advancing microbiological diagnostics.

## Supplementary Information

Below is the link to the electronic supplementary material.

Supplementary file1 (DOCX 1612 KB)

## Data Availability

No datasets were generated or analysed during the current study.
