# Supplementary material for: Magnetic nanoparticle-based method for microorganism concentration in sterile body fluids: Validation and clinical applications
Source: World J Microbiol Biotechnol. 2025 Jul 1;41(7):240. doi: 10.1007/s11274-025-04463-y (PMC12213996; doi:10.1007/s11274-025-04463-y)
Supplement: Supplementary file 1 — Supplementary file1 (DOCX 1612 KB) [file 11274_2025_4463_MOESM1_ESM.docx]

**Supplementary Material**

**Enhanced Concentration of Microorganisms in Clinical Cerebrospinal Fluid Samples Using Magnetic Nanoparticles: Performance, Stability, and Statistical Evaluations**

Bilsen Tural^1,2^*, Erdal Ertaş^2,3^, Nurullah Uzuner^4^, Buşra Bektaş^1^**,** Emre Tural^5^, Mehmet Çavdar^6^, Hakan Temiz^6^, Erdal Özbek^6^, Servet Tural^1,2^

^1^ Department of Nanotechnology, Institute of Science, Dicle University, 21280, Diyarbakir, Turkey

^2^ Department of Chemistry, Institute of Science, Dicle University, 21280, Diyarbakir, Turkey

^3^ Department of Food Processing, Technical Sciences Vocational School, Batman University, Batman, Turkey

^4^25 Aralık State Hospital Şehitkamil, Gaziantep, Turkey

^5^Faculty of Medicine, Department of Child Health and Diseases, Istanbul University-Cerrahpasa, Istanbul, Turkey

^6^Department of Medical Microbiology, Faculty of Medicine, Dicle University, Diyarbakir, Turkey

**Characterizations**

The structural and surface properties of the synthesized IOMNPs were analyzed using advanced characterization techniques, including Transmission Electron Microscopy (TEM (Fig. S1A), Scanning Electron Microscopy (SEM) (Fig. S1B) and Fourier Transform Infrared Spectroscopy (FTIR) (Fig. S3). These analyses confirm the successful synthesis of IOMNPsnanoparticles and their subsequent interactions with microorganisms.

The TEM analysis of IOMNPs, shown in Fig. S1A, reveals the spherical morphology of the nanoparticles with an average particle size ranging between 8 and 10 nm. The high-resolution image indicates the uniformity in particle size and shape, consistent with previous studies utilizing co-precipitation synthesis methods for IOMNPs nanoparticles [19, 27]. Further, the SEM analysis, presented in Fig. S1B, demonstrates the agglomerated nature of the IOMNPsnanoparticles at a larger scale (500 nm). This agglomeration is attributed to the magnetic properties of Fe₃O₄, which causes particle clustering due to magnetic dipole-dipole interactions. Similar observations were reported by Xu et al. [8], where nano-sized IOMNPsparticles exhibited clustering behavior under ambient conditions. Despite agglomeration, the nanoparticles retained their nano-scale dimensions, confirming their suitability for biological applications such as microorganism capture and separation.


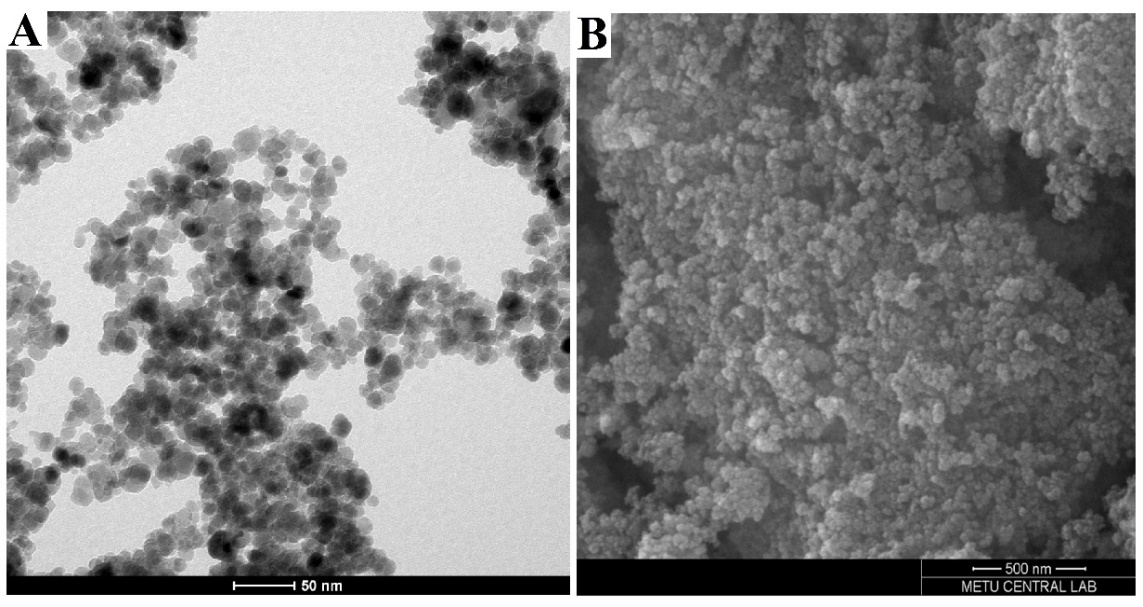


**Figure S1**. A) TEM image of IOMNPs at 50 nm scale; B) SEM image of IOMNPs at 500 nm scale.

FTIR spectroscopy was conducted to identify functional groups on the nanoparticle surface and to evaluate their interactions with microbial species. The FTIR spectrum of pristine IOMNPs (Fig. S2) shows characteristic peaks at 3300 cm⁻¹, corresponding to O-H stretching vibrations, and at 1636 cm⁻¹, associated with adsorbed water bending vibrations. The peak at 535 cm⁻¹ is attributed to the Fe-O bond, confirming the formation of IOMNPs nanoparticles [9, 27].

Following the interaction of IOMNPs with microorganisms, significant changes in the FTIR spectra were observed for Staphylococcus aureus, Enterococcus faecalis, Escherichia coli, Pseudomonas aeruginosa, and Candida albicans (Fig. S2). The increased intensity of the O-H stretching vibration (~3300 cm⁻¹) and adsorbed water bending vibration (~1636 cm⁻¹) suggests hydrogen bonding between microbial hydroxyl groups and the nanoparticle surface [38]. These findings indicate that the microbial surface components, such as hydroxyl groups and water molecules, actively interact with IOMNPs.

Additionally, the appearance of amide I bands (1640–1695 cm⁻¹) in all microorganism-bound spectra highlights the presence of protein-carbonyl interactions, likely originating from bacterial surface proteins [41]. The bands in the range of 1025–1106 cm⁻¹ are attributed to the polysaccharide components of microbial cell walls, specifically the glucosidic bonds. These findings are consistent with the structural composition of microbial cell walls, where polysaccharides and proteins provide binding sites for IOMNPs [42]. The presence of these spectral features confirms the effective attachment of microbial cells to the IOMNPs via both polysaccharide and protein-mediated interactions. The results of the FTIR analysis, supported by TEM and SEM characterization, demonstrate that IOMNPs successfully interact with microorganisms through surface hydroxyl groups, protein carbonyl bonds, and cell wall polysaccharides. These findings provide strong evidence for the feasibility of IOMNPs in the efficient capture and concentration of microorganisms, which is critical for enhancing the sensitivity of diagnostic methods.


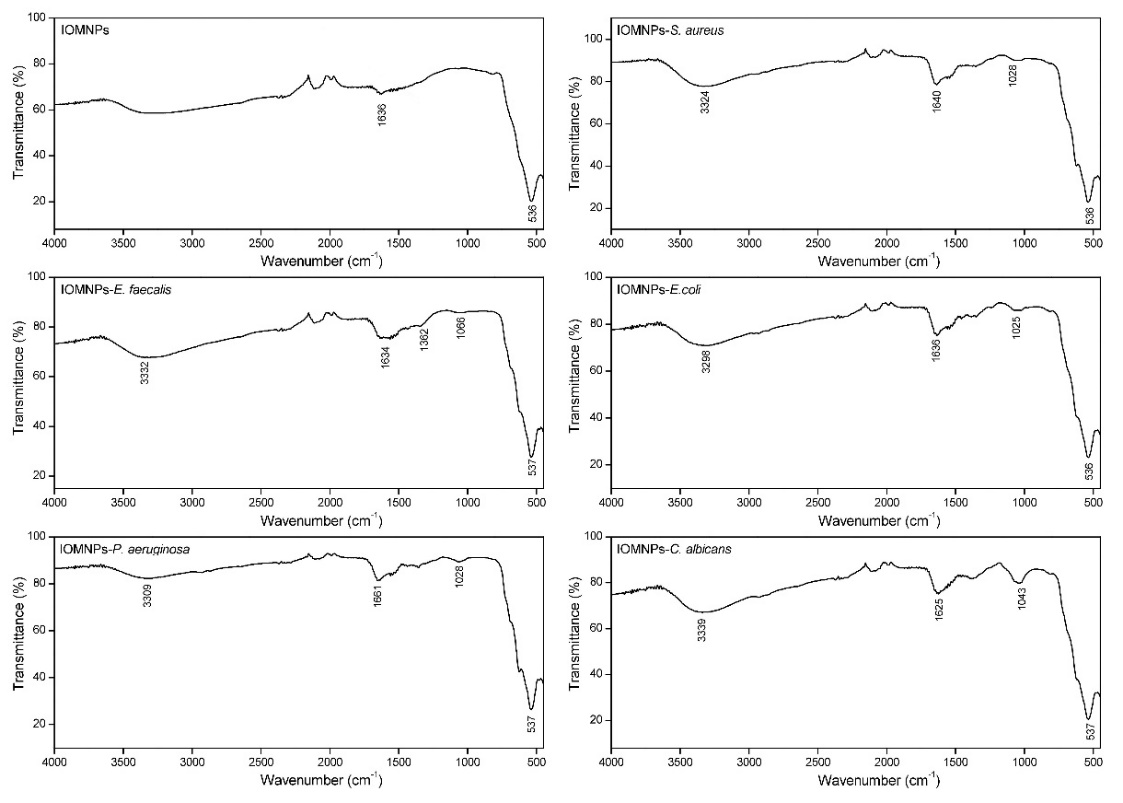


**Figure S2.** FTIR spectra of pristine IOMNPs (IOMNPs) and IOMNPs after interaction with microbial strains (S. aureus, E. faecalis, E. coli, P. aeruginosa, and C. albicans).

**Stability Analyses**


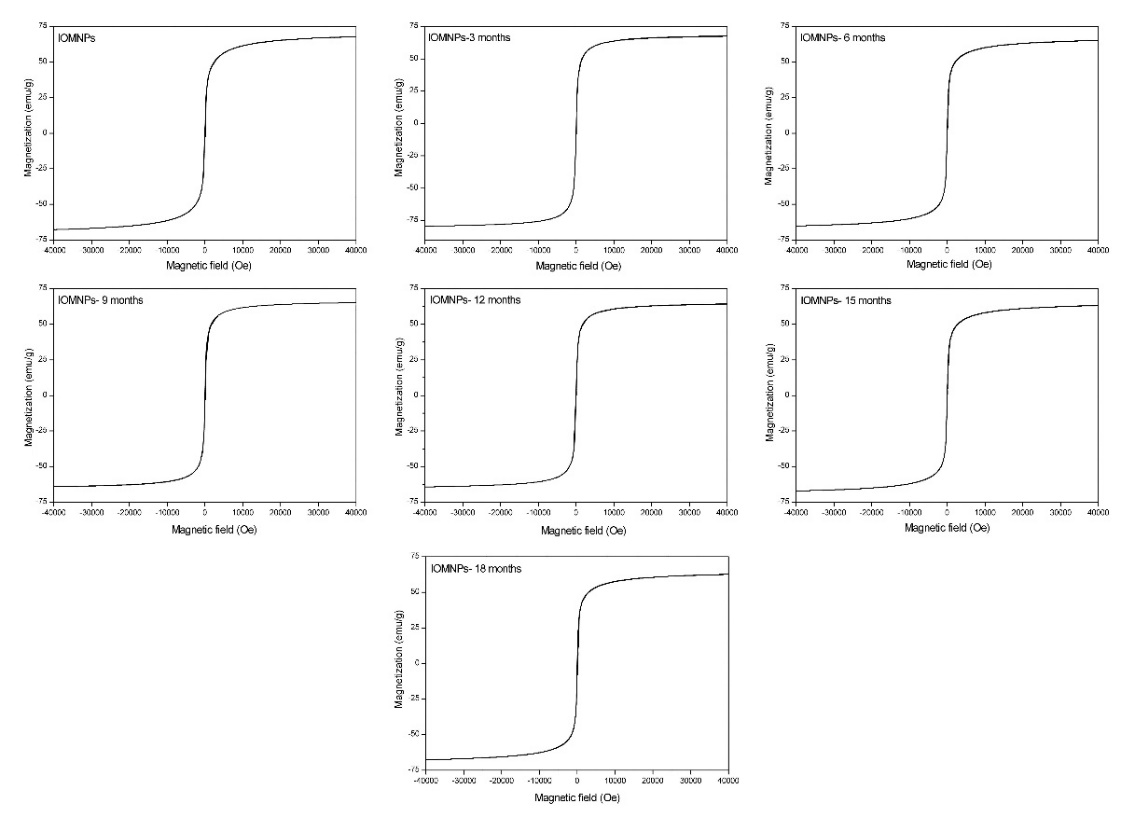


**Figure S3.** M-H Curves of IOMNPs Obtained from Magnetic Stability Analysis at Three-Month Intervals.


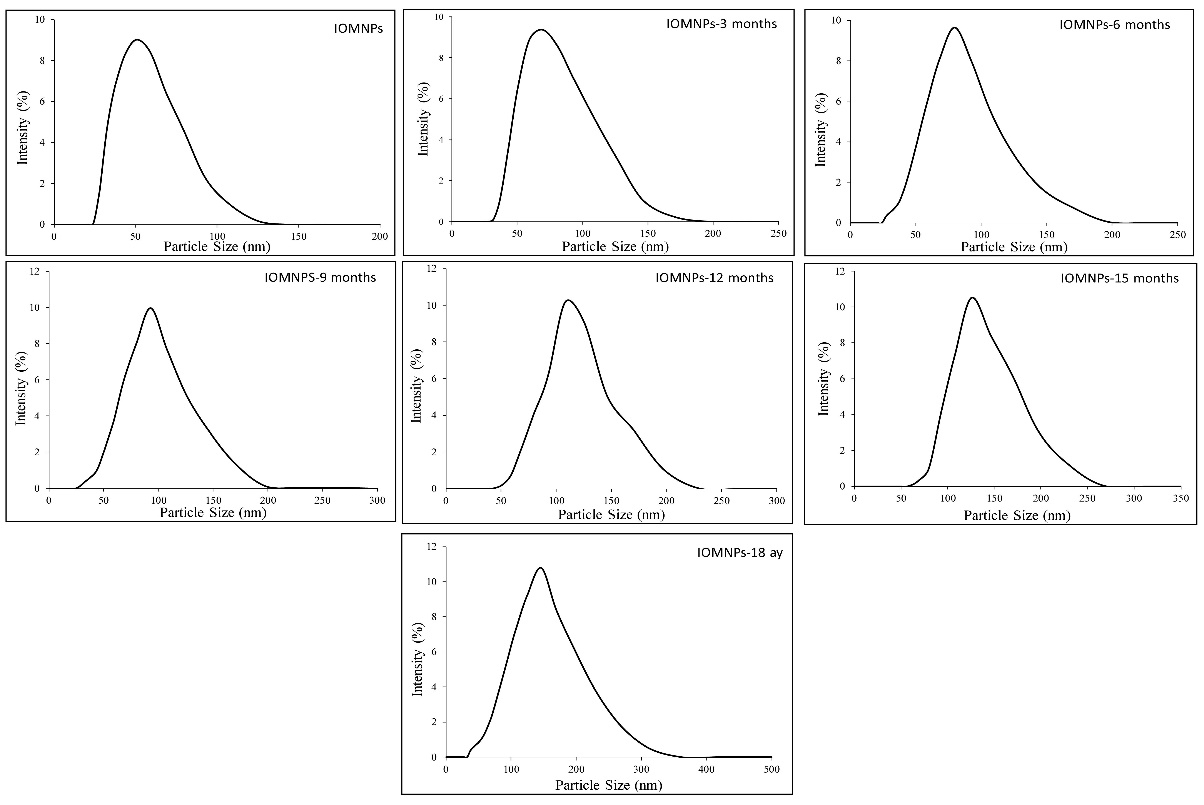


**Figure S4.** Particle Size Distribution Graphs of IOMNPs Obtained from DLS Analysis at Three-Month Intervals.


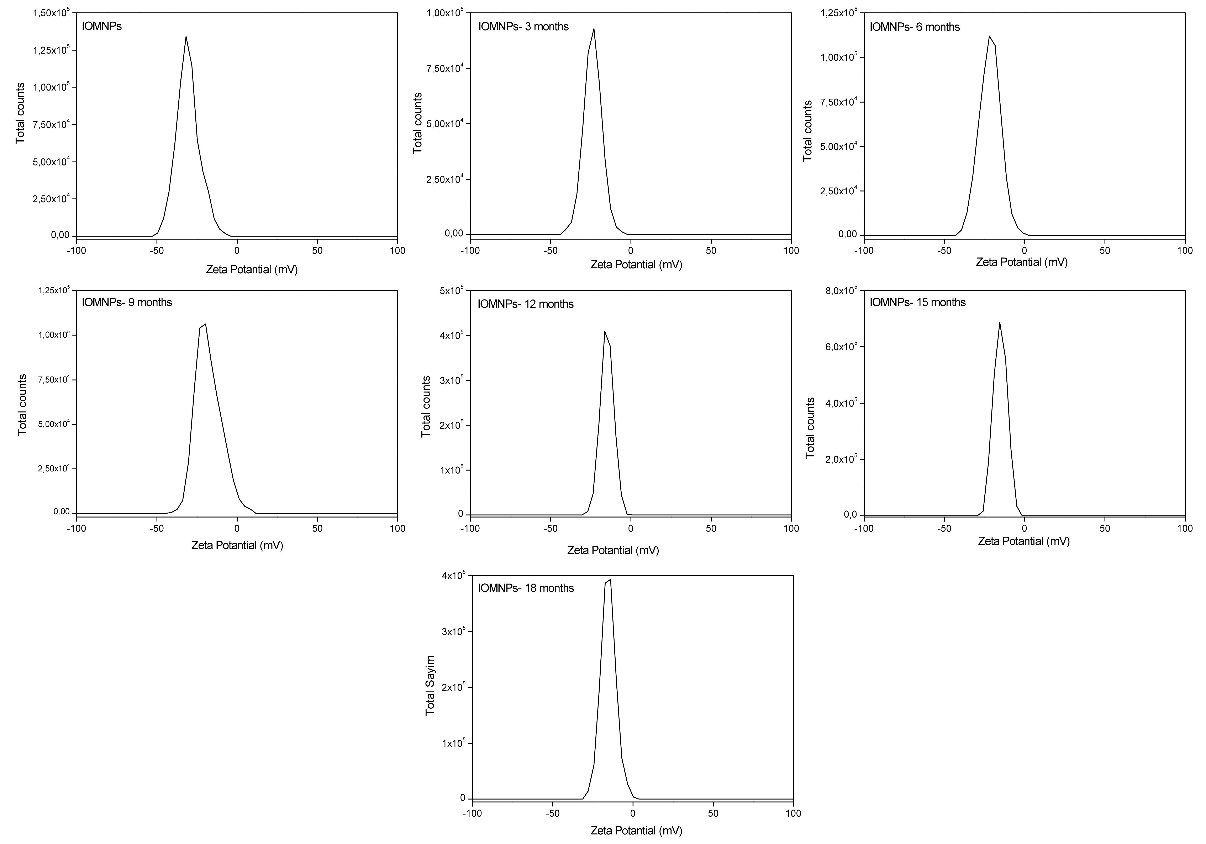


**Figure S5.** Zeta Potential Variation of IOMNPs Measured at Three-Month Intervals.

### **Time and Dosage Optimization Using IOMNPs**

#### **Optimization of Incubation Time**

**Table S1.** Average microbial growth amounts at different incubation durations for IOMNPs-treated microorganisms. *Conditions: 10x10⁷* CFU/mL*, 0.01 g/mL IOMNPs concentration.*

| **Duration (minutes)** | ***S. aureus*** | ***E. faecalis*** | ***E. coli*** | ***P. aeruginosa*** | ***C. albicans*** |
| --- | --- | --- | --- | --- | --- |
| 1 | 10.7±0.6 | 11.3±1.5 | 3.3±0.6 | 22.7±2.1 | 83.3±12.6 |
| 15 | 7.3±0.6 | 10.66±1.2 | 4.7±0.6 | 22.0±1.7 | 91.7±10.4 |
| 30 | 11.7±1.0 | 10.66±1.2 | 5.0±1.0 | 22.0±2.0 | 87.0±6.3 |
| 60 | 11.7±1.0 | 13.3±1.5 | 5.7±1.6 | 13.7±1.2 | 85.0±5.0 |
| 180 | 9.0±1.2 | 18.0±2.7 | 9.7±1.2 | 22.3±2.5 | 66.0±4.6 |

**Table S2.** Average microbial growth amounts at different IOMNPs concentrations. *Conditions: 10x10⁷* CFU/mL*, 1-minute mixing time.*

| **IOMNPs (g/mL)** | ***S. aureus*** | ***E. faecalis*** | ***E. coli*** | ***P. aeruginosa*** | ***C. albicans*** |
| --- | --- | --- | --- | --- | --- |
| 0.007 | 0.3±0.6 | 1.0±0.0 | 0.0±0.0 | 2.0±0.0 | 1.7±2.9 |
| 0.01 | 1.7±0.6 | 1.66±0.6 | 0.7±1.2 | 1.5±0.0 | 2.0±0.0 |
| 0.015 | 1.7±0.6 | 2.0±0.0 | 0.7±1.2 | 2.0±2.0 | 2.3±0.6 |
| 0.020 | 1.3±0.6 | 2.3±0.6 | 1.0±1.7 | 2.0±2.0 | 2.3±2.5 |

These supporting tables provide a detailed comparison of bacterial growth under various conditions, including concentration, duration, and IOMNPs dosage. This information complements the main results, enabling a thorough understanding of the method’s optimization process.

#### **Comparison of Methods for Standard Bacterial Strains**

**Table S3.** Comparative analysis of three methods for microbial concentration across varying McFarland levels (10²–10⁹). Method 3 (IOMNPs -Based Concentration), Method 2 (Centrifugation-Based Concentration), and Method 1 (Standard Plating Method)

| **Concentration**  **(**CFU/mL**)** | ***S. aureus*** | ***E. coli*** | ***E. faecalis*** | ***P. aeruginosa*** | ***C. albicans*** |
| --- | --- | --- | --- | --- | --- |
| **Standard Plating** |  |  |  |  |  |
| 10×10² | 1000±0.0 | 1000±0.0 | 1000±0.0 | 1000±0.0 | 1000±0.0 |
| 10×10³ | 1000±0.0 | 1000±0.0 | 1000±0.0 | 1000±0.0 | 1000±0.0 |
| 10×10⁴ | 1000±0.0 | 1000±0.0 | 1000±0.0 | 1000±0.0 | 200±0.0 |
| 10×10⁵ | 1000±0.0 | 122.0±2.5 | 1000±0.0 | 1000±0.0 | 22±1.0 |
| 10×10⁶ | 34.3±2.1 | 4.00±1.0 | 302±2.0 | 148.66±2.3 | 3.33±0.6 |
| 10×10⁷ | 4.0±1.0 | 0.0±0.0 | 7.66±0.6 | 27.33±0.6 | 0.7±0.6 |
| 10×10⁸ | 0.0±0.0 | 0.0±0.0 | 6.66±0.6 | 3±0.0 | 0.0±0.0 |
| 10×10⁹ | 0.0±0.0 | 0.0±0.0 | 0.0±0.0 | 0.0±0.0 | 0.0±0.0 |
| **Centrifugation-Based Concentration** |  |  |  |  |  |
| 10×10² | 1000±0.0 | 1000±0.0 | 1000±0.0 | 1000±0.0 | 1000±0.0 |
| 10×10³ | 1000±0.0 | 1000±0.0 | 1000±0.0 | 1000±0.0 | 1000±0.0 |
| 10×10⁴ | 1000±0.0 | 1000±0.0 | 1000±0.0 | 1000±0.0 | 221±3.6 |
| 10×10⁵ | 1000±0.0 | 1000±0.0 | 1000±0.0 | 1000±0.0 | 28±1.0 |
| 10×10⁶ | 250.0±5.0 | 101.7±2.1 | 866.7±23.9 | 122.3±2.5 | 1.3±0.6 |
| 10×10⁷ | 35.0±2.0 | 10.0±1.0 | 4.3±0.6 | 18±1.0 | 1.3±0.6 |
| 10×10⁸ | 0.0±0.0 | 0.0±0.0 | 2.0±0.0 | 7±1.0 | 0.0±0.0 |
| 10×10⁹ | 0.0±0.0 | 0.0±0.0 | 0.0±0.0 | 0.0±0.0 | 0.0±0.0 |
| **IOMNPs-Based Concentration** |  |  |  |  |  |
| 10×10² | 1000±0.0 | 1000±0.0 | 1000±0.0 | 1000±0.0 | 1000±0.0 |
| 10×10³ | 1000±0.0 | 1000±0.0 | 1000±0.0 | 1000±0.0 | 1000±0.0 |
| 10×10⁴ | 1000±0.0 | 1000±0.0 | 1000±0.0 | 1000±0.0 | 533.3±29.3 |
| 10×10⁵ | 1000±0.0 | 1000±0.0 | 1000±0.0 | 1000±0.0 | 100±5.0 |
| 10×10⁶ | 283.3±15.3 | 320±98.5 | 1000±0.0 | 616.7±340.3 | 13.3±0.6 |
| 10×10⁷ | 67.7±2.5 | 38.7±6.0 | 145±18.0 | 133.3±58.6 | 13.3±0.6 |
| 10×10⁸ | 2.3±0.6 | 5.0±3.6 | 13.3±4.2 | 10.3±2.5 | 0.0±0.0 |
| 10×10⁹ | 0.0±0.0 | 0.67±0.6 | 2.7±0.6 | 2.3±1.5 | 0.0±0.0 |

**Box Plot Analysis**

The box plot analyses (Fig. S6–S10) visually compare the performance of three methods—Method 1: Standard Plating, Method 2: Centrifugation, and Method 3: DOMNP-based Concentration—for five microorganisms: *Staphylococcus aureus* (*S. aureus*), *Escherichia coli* (*E. coli*), *Enterococcus faecalis* (*E. faecalis*), *Pseudomonas aeruginosa* (*P. aeruginosa*), and *Candida albicans* (*C. albicans*). These plots highlight the distribution, central tendency (median), and variability of the results, providing a clear comparative evaluation of method effectiveness.

For *S. aureus* (Fig. S6), the box plot reveals that *Method 1* has a broad data distribution and the lowest median value, indicating limited efficiency in bacterial recovery. *Method 2* shows improved median values but retains a wide variability, reflecting inconsistent performance. In contrast, *Method 3* (IOMNPs-based method) achieves the highest median with a narrower data spread, demonstrating superior and consistent recovery performance.


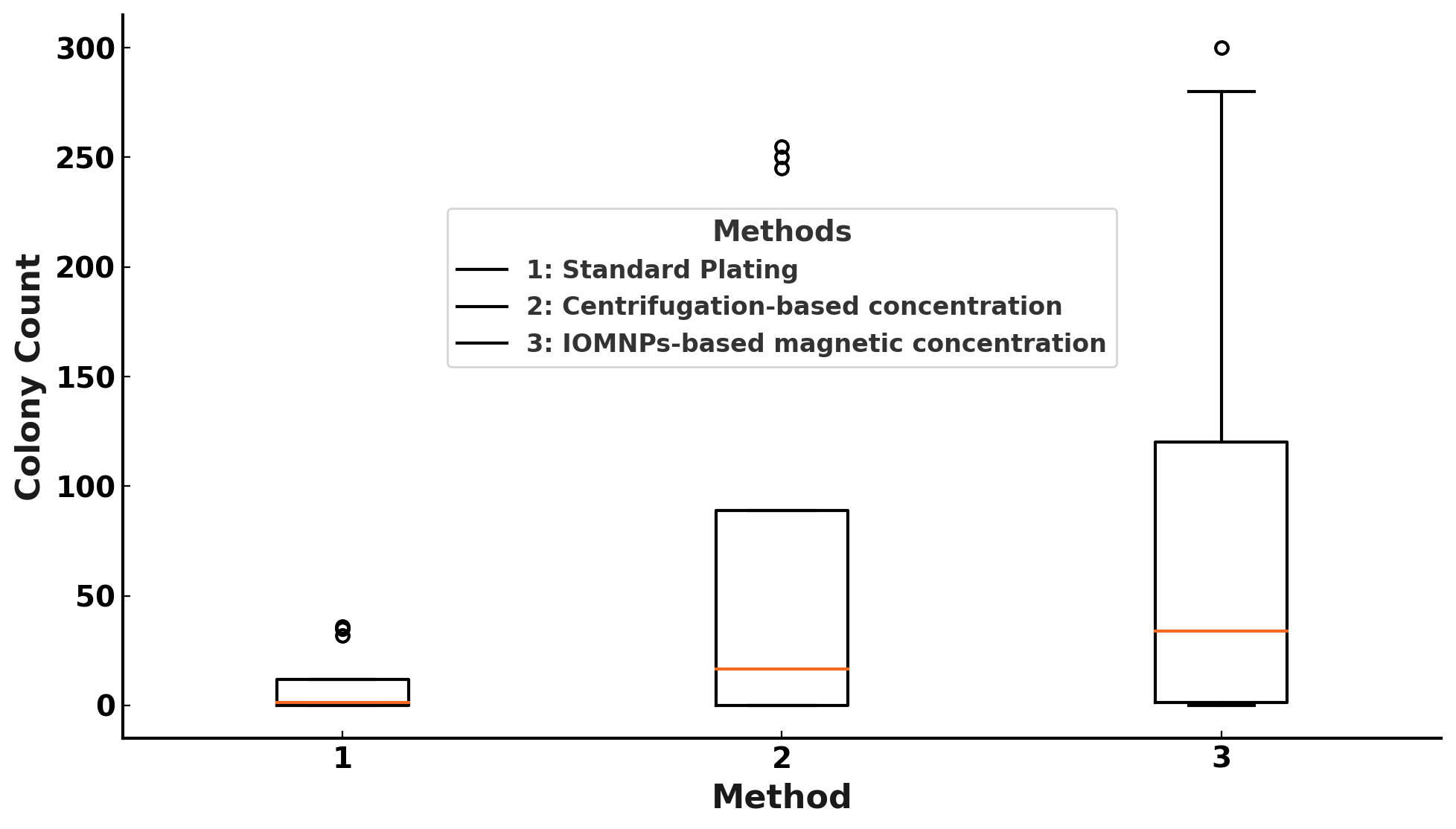


**Figure S6.** Boxplot for *S. aureus*: Distribution of effectiveness for Method 1, Method 2, and Method 3. Method 3 (IOMNPs -Based Concentration), Method 2 (Centrifugation-Based Concentration), and Method 1 (Standard Plating Method)

For *E. coli* (Fig. S7), *Method 1* again exhibits a low median and wide variability, indicating suboptimal performance. *Method 2* achieves higher median values but with irregular data distribution. *Method 3* stands out with the highest median and the most compact distribution, underscoring its effectiveness in concentrating and recovering *E. coli* with minimal variability.


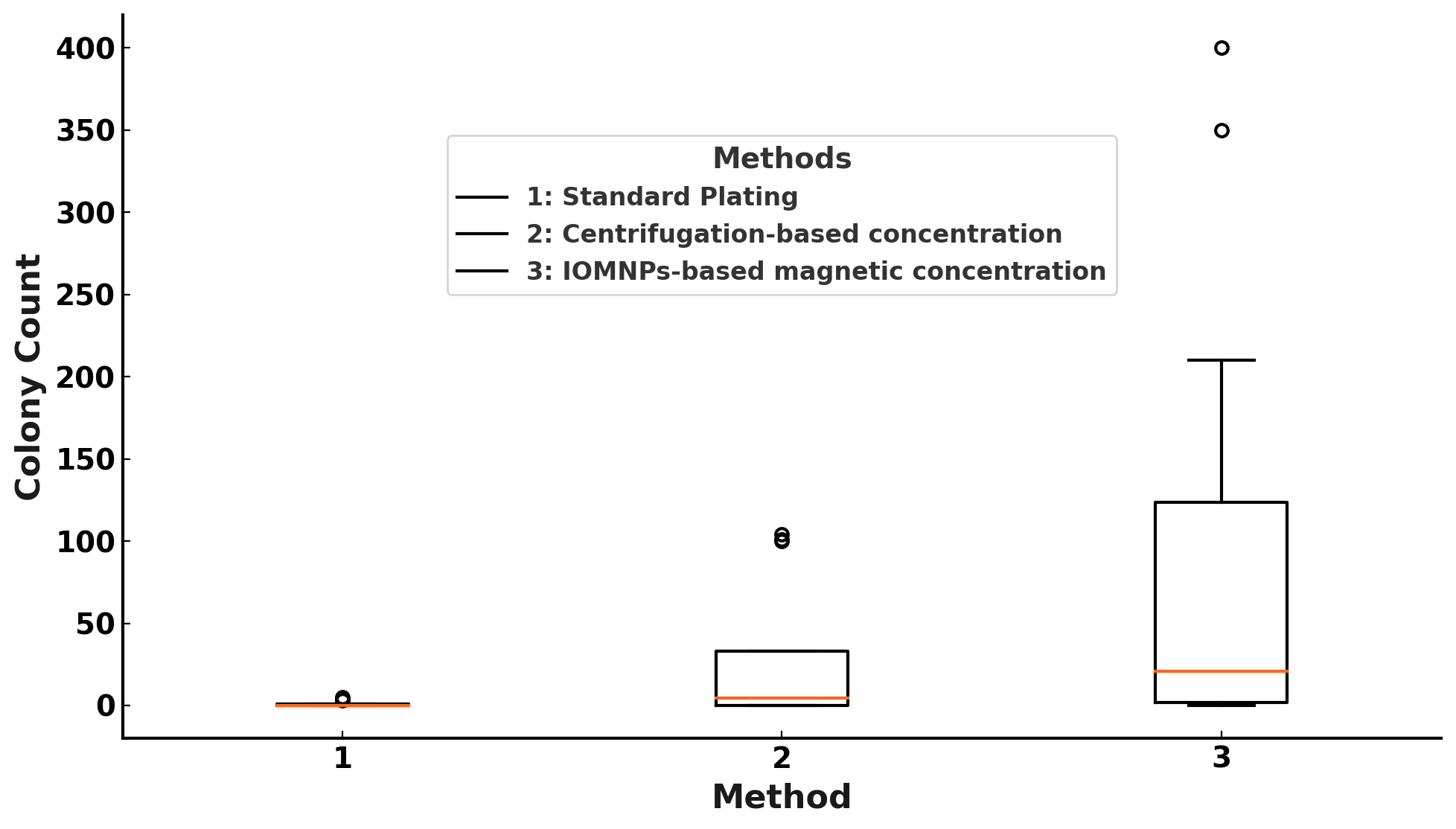


**Figure S7.** Boxplot for *E. coli*: Distribution of effectiveness for Method 1, Method 2, and Method 3. Method 3 (IOMNPs -Based Concentration), Method 2 (Centrifugation-Based Concentration), and Method 1 (Standard Plating Method)

In the case of *E. faecalis* (Fig. S8), *Method 1* shows low median values and a broad spread, suggesting inefficiency. While *Method 2* improves on the median compared to *Method 1*, it still shows significant variability. *Method 3* consistently produces the highest median and a tightly clustered data range, emphasizing its reliability and accuracy in recovering *E. faecalis*.


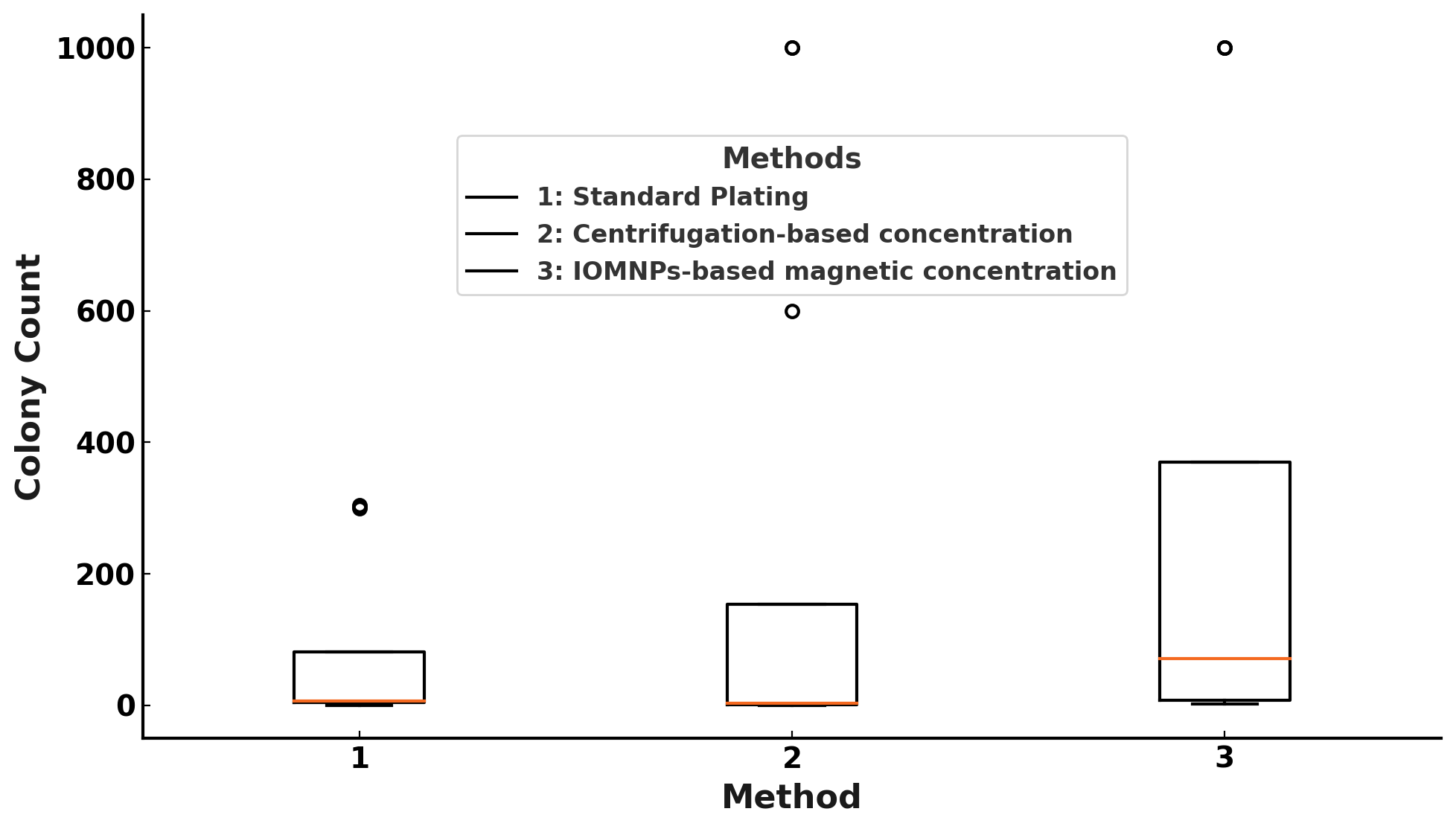


**Figure S8.** Boxplot for *Enterococcus faecalis*: Distribution of effectiveness for Method 1, Method 2, and Method 3. Method 3 (IOMNPs -Based Concentration), Method 2 (Centrifugation-Based Concentration), and Method 1 (Standard Plating Method)

For *P. aeruginosa* (Fig. S9), the data demonstrate that *Method 1* results in a low median and a broad variability, highlighting poor performance. *Method 2* achieves better median values but includes several outliers, reflecting variability. In contrast, *Method 3* achieves the highest median with the narrowest spread, indicating its robustness and efficiency in concentrating *P. aeruginosa*.


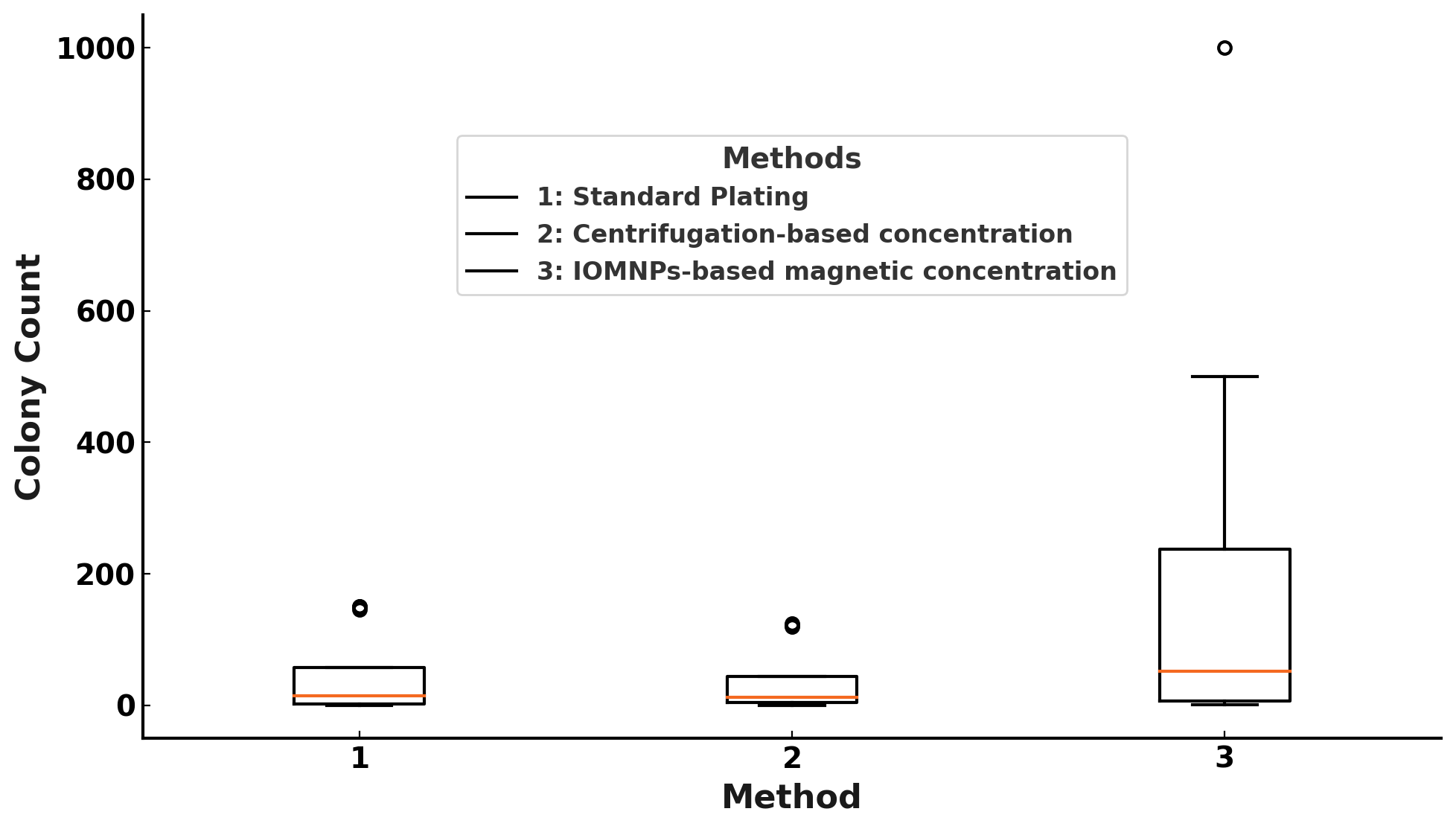


**Figure S9.** Boxplot for *Pseudomonas aeruginosa*: Distribution of effectiveness for Method 1, Method 2, and Method 3. Method 3 (IOMNPs -Based Concentration), Method 2 (Centrifugation-Based Concentration), and Method 1 (Standard Plating Method)

For *C. albicans* (Fig. S10), the trends remain consistent. *Method 1* exhibits a low median and wide data spread, demonstrating reduced effectiveness. *Method 2* improves slightly in terms of median values but shows inconsistent results. *Method 3* achieves the highest median with a compact distribution, confirming its enhanced performance in recovering *C. albicans*.

**
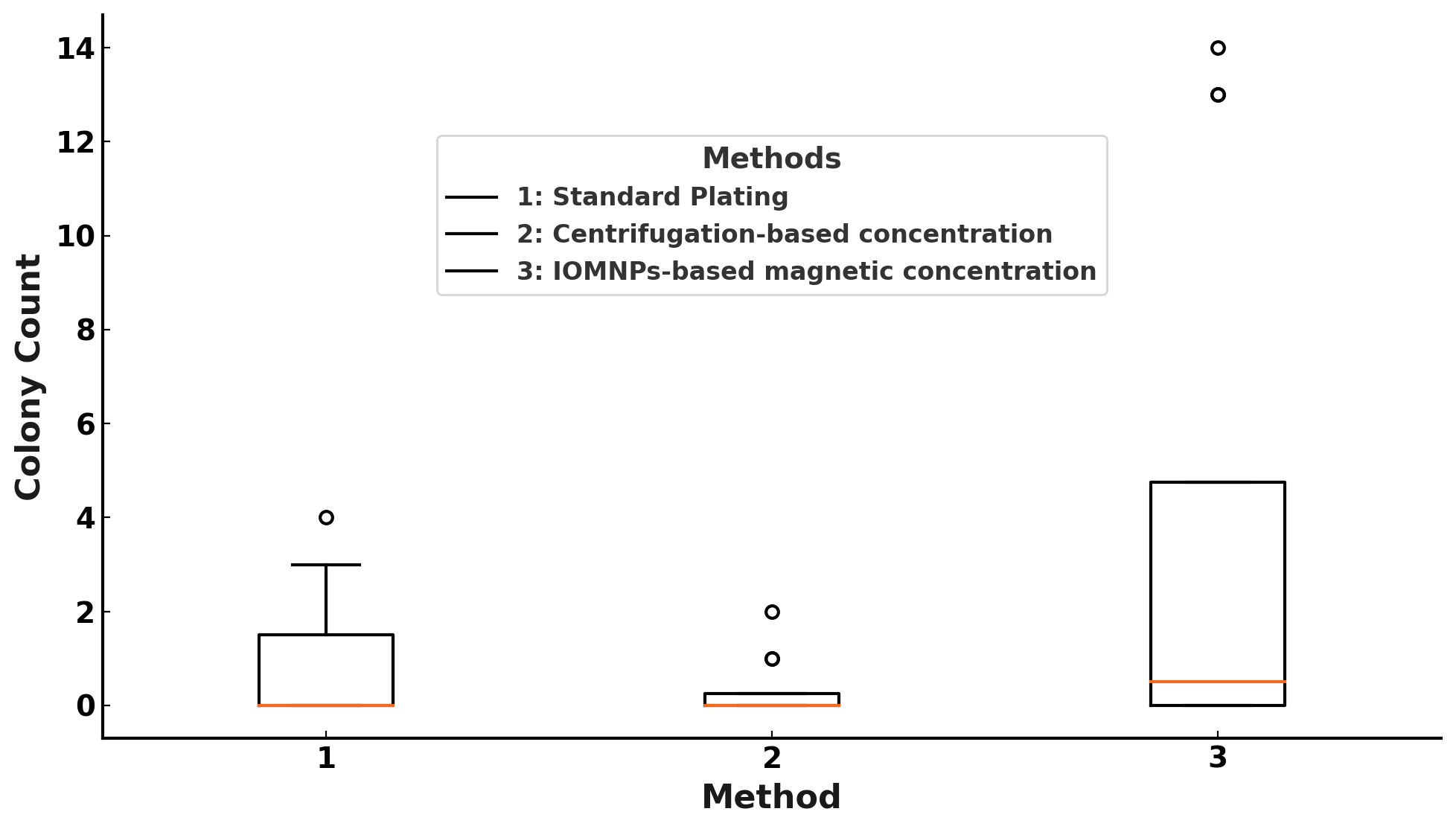
**

**Figure S10.** Boxplot for *Candida albicans*: Distribution of effectiveness for Method 1, Method 2, and Method 3. Method 3 (IOMNPs -Based Concentration), Method 2 (Centrifugation-Based Concentration), and Method 1 (Standard Plating Method)

Across all five microorganisms, *Method 3* (IOMNPs-based concentration) consistently outperforms *Method 1* and *Method 2*. The higher medians and narrower data spreads indicate that IOMNPs provide reliable, accurate, and reproducible results, particularly for low-density microbial suspensions. This superiority aligns with literature findings that magnetic nanoparticle-based techniques offer enhanced sensitivity and precision for microbial concentration [32].

**Limit of Detection (LOD) For IOMNPs-Based Concentration Method**

**Table S4.** Average microbial growth amounts for various bacterial concentrations using IOMNPs. *Conditions: 0.01 g/mL IOMNPs-based concentration, 1-minute mixing time.*

| **Concentration (CFU/mL)** | ***S.aureus*** | ***E.faecalis*** | ***E.coli*** | ***P.aeruginosa*** | ***C.albicans*** |
| --- | --- | --- | --- | --- | --- |
| 10x10²- | 1000±0.0 | 1000±0.0 | 1000±0.0 | 1000±0.0 | 1000±0.0 |
| 10x10³- | 1000±0.0 | 1000±0.0 | 1000±0.0 | 1000±0.0 | 1000±0.0 |
| 10x10⁴- | 1000±0.0 | 1000±0.0 | 1000±0.0 | 1000±0.0 | 491.7±7.5 |
| 10x10⁵- | 1000±0.0 | 1000±0.0 | 1000±0.0 | 1000±0.0 | 129.3±5.1 |
| 10x10⁶- | 283.3±15.3 | 1000±0.0 | 385±15.0 | 400.0±10.0 | 11.3±1.5 |
| 10x10⁷- | 67.7±2.5 | 488.3±10.4 | 37.0±3.6 | 103.3±7.6 | 1.3±0.6 |
| 10x10⁸- | 2.3±0.57 | 386.7±15.3 | 4±1.0 | 8.8±1.2 | 0.0±0.0 |
| 10x10⁹- | 0 | 2.3±0.6 | 1.3±0.6 | 2.7±1.2 | 0.0±0.0 |

#### **Pathogen Identification in CSF Samples**


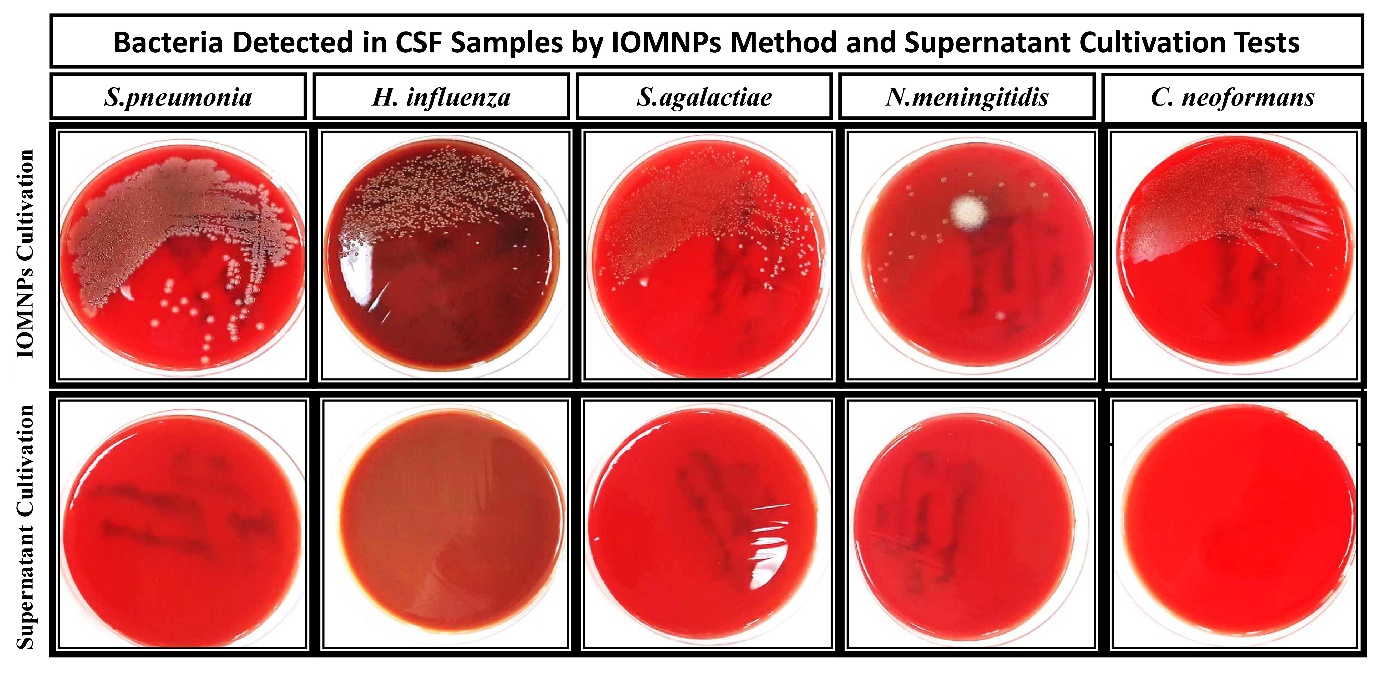


**Figure S11.** Identification of microorganisms in CSF samples using the IOMNPs-based concentration method and their supernatant growth results.
